# Identification of a CCR5-Expressing T Cell Subset That Is Resistant to R5-Tropic HIV Infection

**DOI:** 10.1371/journal.ppat.0030058

**Published:** 2007-04-27

**Authors:** Kyra Oswald-Richter, Stacy M Grill, Mindy Leelawong, Michelle Tseng, Spyros A Kalams, Todd Hulgan, David W Haas, Derya Unutmaz

**Affiliations:** 1 Department of Microbiology and Immunology, Vanderbilt University Medical School, Nashville, Tennessee, United States of America; 2 Division of Infectious Diseases and Department of Medicine, Vanderbilt University Medical School, Nashville, Tennessee, United States of America; King's College London, United Kingdom

## Abstract

Infection with HIV-1 perturbs homeostasis of human T cell subsets, leading to accelerated immunologic deterioration. While studying changes in CD4^+^ memory and naïve T cells during HIV-1 infection, we found that a subset of CD4^+^ effector memory T cells that are CCR7^−^CD45RO^−^CD45RA^+^ (referred to as T_EMRA_ cells), was significantly increased in some HIV-infected individuals. This T cell subset displayed a differentiated phenotype and skewed Th1-type cytokine production. Despite expressing high levels of CCR5, T_EMRA_ cells were strikingly resistant to infection with CCR5 (R5)–tropic HIV-1, but remained highly susceptible to CXCR4 (X4)–tropic HIV-1. The resistance of T_EMRA_ cells to R5-tropic viruses was determined to be post-entry of the virus and prior to early viral reverse transcription, suggesting a block at the uncoating stage. Remarkably, in a subset of the HIV-infected individuals, the relatively high proportion of T_EMRA_ cells within effector T cells strongly correlated with higher CD4^+^ T cell numbers. These data provide compelling evidence for selection of an HIV-1–resistant CD4^+^ T cell population during the course of HIV-1 infection. Determining the host factors within T_EMRA_ cells that restrict R5-tropic viruses and endow HIV-1–specific CD4^+^ T cells with this ability may result in novel therapeutic strategies against HIV-1 infection.

## Author Summary

HIV-1 infection profoundly perturbs the immune system and is characterized by depletion of CD4^+^ T cells and chronic immune activation, which lead to AIDS. Although HIV-1 targets CD4^+^ T cells, it also requires a second receptor in order to infect the target cells. The majority of HIV-1 strains that are transmitted use a cell surface molecule called CCR5, which is expressed on a portion of T cells. In this manuscript we identify a subset of human CD4^+^ T cells, which we termed T_EMRA_ cells, that express CCR5 but still remain resistant to infection. We show that HIV-1 infection is blocked in T_EMRA_ cells after entry of the virus, but before it has a chance to integrate into the cellular genome. T_EMRA_ cells are present at low frequency in HIV-1 uninfected individuals but greatly increase in some HIV-infected individuals, which correlates with higher CD4^+^ T cell numbers. These findings provide the basis for future studies to understand the role of T_EMRA_ cells during HIV-1 infection and identify the host factors that could restrict the virus. This knowledge may be used to endow susceptible T cells with the ability to resist infection and result in novel vaccine or therapeutic strategies against HIV-1 infection.

## Introduction

Chronic immune activation and homeostatic disturbance of T cell subsets that accompany viral replication are hallmarks of HIV-1 infection [1–4]. The cause and implications of these profound quantitative and qualitative changes in CD4^+^ memory T cell subsets during HIV-1 infection are still not well understood [2]. Elucidating the causal relationships between perturbed naïve and memory T cell compartments during the course of HIV-1 infection could be critical in understanding its pathogenesis.

Human T cells are categorized as naïve (T_N_) and memory (T_M_) subsets based on expression of CD45RA and CD45RO isoforms, respectively [5–8]. It is now known that memory T cells are comprised of distinct subsets that can be identified based on other surface markers and effector functions [9]. Sallusto and colleagues defined two CD4^+^ memory T cell subsets, termed central memory (T_CM_) and effector memory (T_EM_) cells [8]. T_EM_ cells have low expression levels of the chemokine receptor CCR7 and lymph node homing receptor CD62L, express receptors for migration to inflamed tissues, and display immediate effector functions [8,10]. In contrast, T_CM_ cells express high levels of CCR7 and lack potent effector functions. It has been proposed that T_CM_ cells are responsible for maintaining long-term memory, and upon re-exposure to antigens, differentiate into T_EM_ cells with effector functions. Prior studies indicated that HIV-1 preferentially infects memory, rather than naïve CD4^+^ T cells [11–16], possibly because of exclusive expression of the HIV-1 coreceptor CCR5 on memory T cells. Within the memory population, T_EM_ cells are enriched for expression of CCR5 relative to other CD4 memory cells [17,18], suggesting that they may be primary targets for CCR5-tropic (R5-tropic) viruses that predominate in most infected persons.

Because chronic HIV-1 infection disrupts the balance between naïve and memory T cell subsets [19], we characterized the distribution of these cells during HIV-1 infection. We found that a small subset of CD4^+^ T_EM_ cells, which we called CD4^+^ T_EMRA_ cells, were greatly increased in some HIV-infected individuals relative to uninfected individuals. Remarkably, CD4^+^ T_EMRA_ cells displayed a specific post-entry block to R5-tropic HIV-1, despite expressing high levels of CCR5. Accumulation of this effector memory CD4^+^ T cell subset during chronic HIV infection could have important implications in understanding intrinsic resistance to the virus and perturbation of T cell compartments in infected individuals.

## Results

### Human T Cell Subset Distribution during HIV-1 Infection

**Figure 1:**
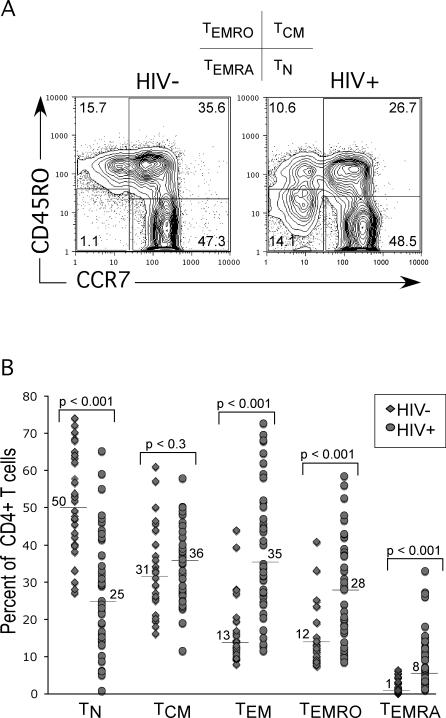
Distribution of Human CD4^+^ T Cell Subsets in HIV-Infected and HIV-Uninfected Individuals (A) PBMCs from HIV-uninfected and HIV-infected individuals were stained with purified CCR7 antibody, followed by allophycocyanin-conjugated anti-mouse IgG. After washing, cells were co-stained with CD4-FITC, CD3-Percp.Cy5.5, and CD45RO-PE. Expression of CCR7 and CD45RO were analyzed after gating on CD4^+^CD3^+^ T cells. Representative flow cytometry data from over 60 individuals are shown. The abbreviated definition of T cell subsets based on this staining profile is shown above the flow cytometry plots. (B) CD4^+^ T cell subsets were analyzed in HIV-uninfected and HIV-infected individuals. Between 300,000 and 500,000 events were collected for each sample, and electronic gates were set on CD4^+^CD3^+^ T cells. Distribution and median percentages of T_N_ (CD45RO^−^CCR7^+^), T_CM_ (CD45RO^+^CCR7^+^), T_EMRO_ (CD45RO^+^CCR7^−^), and T_EMRA_ (CD45RO^−^CCR7^−^) cells in 33 HIV-infected and 30 HIV-uninfected individuals are shown.

The dynamics of T cell changes were studied in HIV-infected and HIV-uninfected individuals by staining their peripheral blood mononuclear cells (PBMCs) with monoclonal antibodies against CD3, CD4, CCR7, and CD45RO cell surface molecules. In most uninfected individuals, this analysis divides CD4^+^ T cells into three subsets that can be readily quantified: naïve T cells (T_N_; CD45RO^−^CCR7^+^), central memory T cells (T_CM_; CD45RO^+^CCR7^+^), and effector memory T cells (T_EM_; CCR7^−^) (Figure 1A, left panel). However, in HIV-uninfected individuals, a fourth subset (CD45RO^−/dull^CCR7^−^) was also observed (Figure 1A), albeit with a low frequency (0.5%–3%). This subset was greatly increased in some of the HIV-infected individuals (Figure 1A, right panel). Because these cells resembled a previously defined CD8^+^ T cell subset (called CD8^+^ T_EMRA_ cells) with effector functions that expressed CD45RA with effector functions [20], we tentatively termed them CD4^+^ T_EMRA_ cells (referred to as T_EMRA_ cells hereafter). Conversely, we denoted the CD45RO^+^CD45RA^−^CCR7^−^ effector memory CD4^+^ T cell subset as T_EMRO_ cells. The relationship between T_EMRA_ cells and HIV-1 infection was studied in 33 HIV-infected and 30 HIV-uninfected individuals (Figure 1B). The proportion of T_EMRO_ and T_EMRA_ subsets was significantly increased in HIV-infected individuals (Figure 1B). On the other hand, the proportion of T_N_ cells was significantly decreased in HIV-infected individuals, while the proportion of T_CM_ cells remained similar in both groups (Figure 1B).

### Phenotypic Characterization of CD4^+^ T_EMRA_ Cells

**Figure 2:**
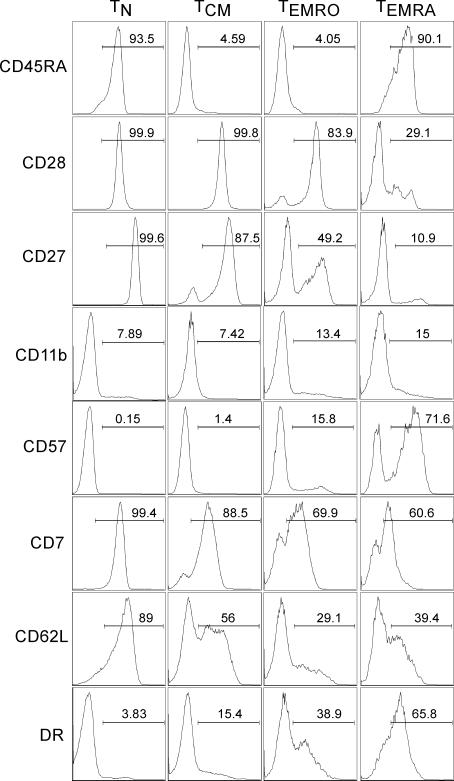
Phenotype of Distinct CD4^+^ Human T Cell Subsets Purified CD4^+^ T cells were stained with CD45RO and CCR7 in conjunction with the antibodies shown. Electronic gates were set on T_N_, T_CM_, T_EMRO_, and T_EMRA_ cells as described in Figure 1A, and expression of various T cell surface markers was analyzed. A representative profile from an HIV-infected individual is shown. Similar phenotype was observed in all four subsets of T cells from six HIV-infected and HIV-uninfected individuals.

The high proportion of T_EMRA_ cells found in HIV-infected individuals prompted further analysis of this subset. We hypothesized that T_EMRA_ cells are a subset of effector memory CD4^+^ T cells, analogous to a subset recently described for CD8^+^ T cells with the same surface marker phenotype [20]. The four subsets of CD4^+^ T cells (T_N_, T_CM_, T_EMRO_, and T_EMRA_) obtained from HIV-infected and HIV-uninfected individuals were analyzed for expression of cell surface molecules known to be expressed differentially in naïve, memory, and effector T cells. All T_N_ cells expressed CD28, CD27, CD7, and CD62L, with progressively less expression on CD4^+^ T_CM_, T_EMRO_, and T_EMRA_ cells (Figure 2). In contrast, expression of CD11b, CD57, and HLA-DR were increased on T_EMRO_ and T_EMRA_ cells compared to CD4^+^ T_N_ and T_CM_ cells (Figure 2). In contrast to T_CM_ and T_EMRO_ cells, T_EMRA_ cells also expressed high levels of CD45RA, similar to T_N_ cells (Figure 2, top panel). This profile suggested that T_EMRA_ cells are a subset of CD4^+^ effector memory T cells with a peculiar CD45RA^+^CD45RO^−/dull^ phenotype.

### Proliferative Capacity and Apoptosis of Effector Memory T Cell Subsets

**Figure 3:**
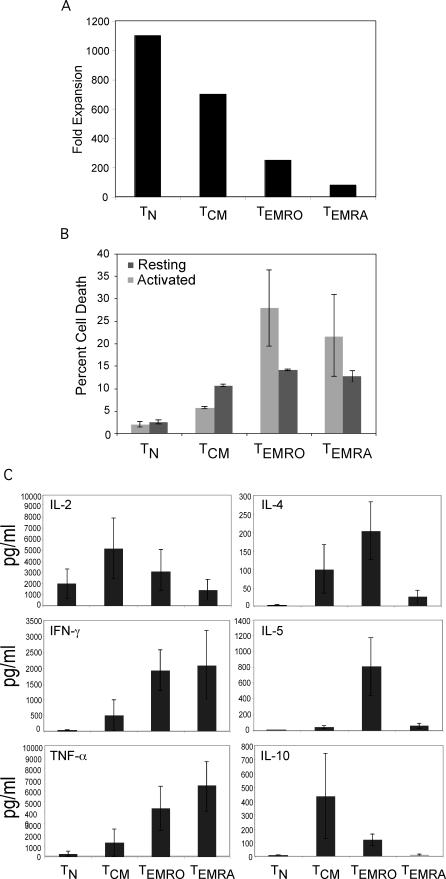
Proliferation, Apoptosis, and Cytokine Profile of CD4^+^ T Cell Subsets (A) Sorted CD4^+^ T cell subsets from an HIV-uninfected individual were activated using DCs pulsed with SEB (10 ng/ml) and expanded in IL-2–containing medium for 12 d. T cell subsets were then counted to determine fold expansion. (B) Viability of purified CD4^+^ T cell subsets was determined using Annexin V staining after 18 h post-activation. Samples were analyzed by flow cytometry. Statistical significance was determined using the Student's two-tailed *t* test. T_N_: *p* = 0.4, T_CM_: *p* = 0.002, T_EMRO_: *p* = 0.15, and T_EMRA_: *p* = 0.3. (C) Sorted CD4^+^ T cell subsets were activated using plate-bound anti-CD3 (3 μg/ml) and soluble anti-CD28 (1 μg/ml) antibodies. Supernatants were collected 18–24 h post-activation and analyzed for cytokine production using the cytometric bead assay. The results show the mean of four different experiments from four different individuals.

Differentiated effector memory T cells have a reduced proliferative capacity [8,20]. To assess the relative proliferative capacity of the different CD4^+^ T cell subsets, each subset was purified from an HIV-uninfected individual according to CCR7 and CD45RO expression as shown in Figure 1A, and stimulated with dendritic cells (DCs) pulsed with superantigen (staphylococcal enterotoxin B [SEB]). Activated T cells were counted at day 12 (Figure 3A). DC-mediated activation caused robust cell division of T_N_ and T_CM_ cells (Figure 3A), whereas T_EMRO_ cells and T_EMRA_ cells divided fewer times (Figure 3A).

The reduced proliferative capacity of effector T cells correlates with a decrease in telomere length and with an increased propensity to undergo apoptosis [9]. To determine whether T_EMRA_ cells undergo apoptosis similar to effector T cells, all T cell subsets were stained with a marker of apoptosis (Annexin V) before and after cells were stimulated through the T cell receptor (TCR) by anti-CD3 plus anti-CD28 antibodies for 18 h. A higher proportion of effector T cells underwent apoptosis compared to T_N_ and T_CM_ cells (Figure 3B). Levels of apoptosis were comparable between T_EMRO_ and T_EMRA_ cells before and after TCR stimulation (Figure 3B).

### Cytokine Profile of Human Effector Memory T Cells

A hallmark of T_EM_ cells is secretion of greater quantities of cytokines when stimulated through the TCR, as compared to T_N_ and T_CM_ cells [8,10]. We therefore explored cytokine profiles of T_EMRO_ and T_EMRA_ subsets. As expected, [8,10] T_EMRO_ cells secreted greater amounts of most cytokines assayed (IL-4, IL-5, IL-10, TNF-α, and IFN-γ) compared to T_N_ and T_CM_ cells (Figure 3C). T_EMRA_ cells secreted high levels of IFN-γ, but much lower levels of IL-4, IL-5, or IL-10 compared to T_EMRO_ cells (Figure 3C). This cytokine profile suggested that the T_EMRA_ subset is skewed towards a Th1 phenotype. Recently, a cell surface molecule called CRTH2 was shown to be highly expressed on Th2 but not on Th1 cells [21]. To confirm Th1 skewing of T_EMRA_ cells, we analyzed the surface expression of CRTH2. In agreement with the cytokine profile, significantly fewer T_EMRA_ cells expressed CRTH2 compared to T_EMRO_ or T_CM_ subsets (Figure S1). Taken together, we conclude that T_EMRA_ cells are differentiated effector memory T cells that are skewed toward a Th1 phenotype.

### HIV-1 Infection of TCR-Stimulated CD4^+^ T Cell Subsets

Because T_EMRA_ cells were proportionately increased in some HIV-infected individuals, we next investigated the susceptibility of these cells to HIV-1 infection. For these experiments, T_N_, T_CM_, T_EMRO_, and T_EMRA_ cells purified from PBMCs of HIV-infected and HIV-uninfected individuals were activated through the TCR to render them susceptible to infection. The activated T cells were then infected with either R5-tropic replication-competent HIV (R5.HIV), CXCR4 (X4)–tropic replication-competent HIV (X4.HIV), or replication-defective viruses that only undergo a single round of replication and are pseudotyped with vesicular stomatitis virus glycoprotein G (VSV-G.HIV). Each virus used here encoded green fluorescent protein (GFP) that was used to quantify infection by flow cytometry at specific time points after inoculation [22].

**Figure 4:**
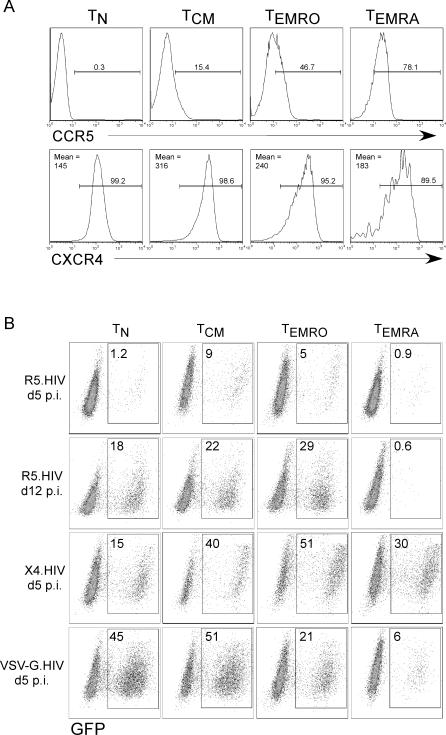
Expression of HIV-1 Coreceptors on CD4^+^ T Cell Subsets and Their Susceptibility to HIV-1 Infection (A) CD4^+^ T cells from an HIV-uninfected individual were stained with CD45RO-PE and CCR7-FITC in conjunction with CCR5 and CXCR4 antibodies. CD4^+^ T cell subsets were gated as described in Figure 1, and expression of CCR5 and CXCR4 was analyzed. (B) Purified CD4^+^ T cell subsets from an HIV-uninfected individual were activated using plate-bound anti-CD3 (3 μg/ml) and soluble anti-CD28 (1 μg/ml) antibodies and concurrently infected with VSV-G.HIV, R5.HIV, or X4.HIV. Percent infected cells was determined by GFP expression of T cells on day 5 and 12 post-infection (p.i.). The results are representative of one of five independent experiments using T cell subsets isolated from five different individuals. The median infection values at day 5 were: R5-HIV: T_N_ = 1.1, T_CM_ = 9.5, T_EMRO_ = 5.4, and T_EMRA_ = 0.85; X4-HIV: T_N_ = 17, T_CM_ = 36, T_EMRO_ = 43, and T_EMRA_ = 33; and VSV-G.HIV: T_N_ = 44, T_CM_ = 48, T_EMRO_ = 19, and T_EMRA_ = 6.5.

Prior to the infectivity assay, we analyzed the expression of HIV-1 co-receptors CCR5 and CXCR4 on T_N_, T_CM_, T_EMRO_, and T_EMRA_ cells isolated from an HIV-uninfected individual (Figure 4A). T_EMRA_ and T_EMRO_ cells expressed the highest levels of CCR5, while all four subsets expressed high levels of CXCR4 (Figure 4A). In addition, the median CCR5 expression was quantitated from 20 HIV-infected individuals, and the similar subset expression trends were confirmed (Figure S2). When each T cell subset isolated from an HIV-uninfected individual was challenged with R5.HIV, CD4 T_CM_ and T_EMRO_ cells were more susceptible to infection than T_N_ cells (Figure 4B), most likely reflecting high CCR5 surface expression levels on these memory T cells (Figure 4A). In contrast, T_EMRA_ cells were resistant to a high multiplicity challenge with R5.HIV (Figure 4B, top panel). This was an unexpected finding given the high cell surface CCR5 levels on T_EMRA_ cells (Figure 4A). At day 12 post-infection, R5.HIV spread through the cultures, producing more infected T_N_, T_CM_, and T_EMRO_ cells as compared to 5 d post-infection. Even at this late time point, T_EMRA_ cells remained almost completely refractory to infection (Figure 4B, second panel). In contrast, T_EMRA_ cells were similarly susceptible to infection with X4.HIV, as well as other T cell subsets (Figure 4B, third panel). Surprisingly, T_EMRA_ cells were also 5- to 10-fold less susceptible to VSV-G.HIV infection than other T cell subsets (Figure 4B, bottom panel).

**Figure 5:**
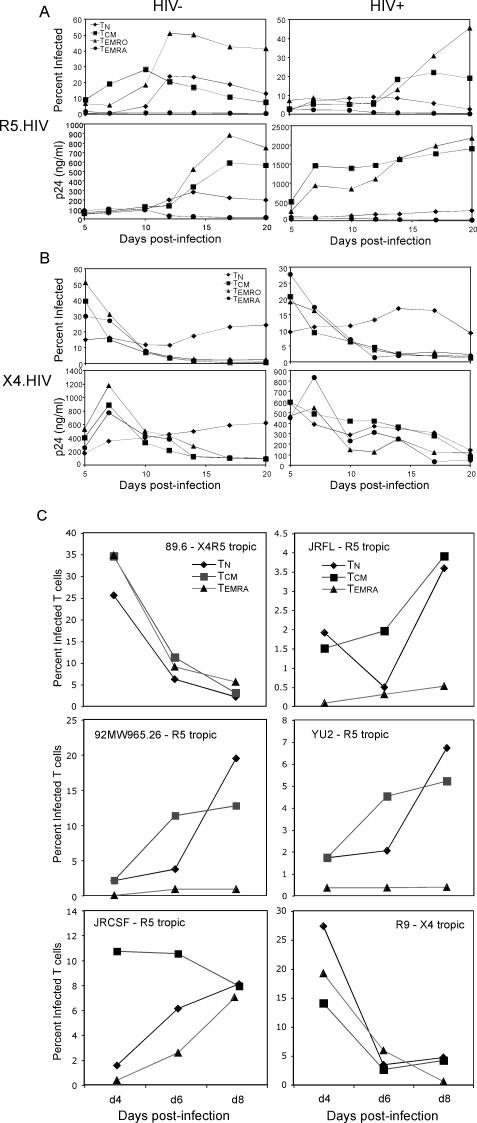
Replication of HIV-1 Strains in CD4^+^ T Cell Subsets Purified CD4^+^ T cell subsets were activated through TCR as in Figure 4 and concurrently infected with replication-competent HIV-1. (A) R5.HIV and X4.HIV infection time course of CD4^+^ T cell subsets from HIV-uninfected and HIV-infected individuals. (B) Supernatants from purified CD4^+^ T cell subsets infected with R5.HIV and X4.HIV cultures were collected at different time points and HIV p24 levels were measured by ELISA. The percentage of infected cells was determined by GFP expression at different time points post-infection by flow cytometry. The results are representative of one of five independent experiments from different individuals. (C) Infection of T cell subsets with HIV-1 expressing different primary isolate envelopes. Percent infected T cells was determined by intracellular p24 staining of T cells on day 4, 6, and 8 post-infection. The T cell subsets in this experiment were also infected with VSG-V.HIV, where the percent infected cells was determined by GFP expression at 4 day post-infection. The VSV-G-HIV infection was 33% for T_N_, 33% for T_CM_, and 9% for T_EMRA_ cells.

We then sought to determine whether over time the T_EMRA_ subset would progressively become more susceptible to infection post-activation, or whether these cells were being killed in culture by rapidly replicating virus. For this experiment, T cells were infected with R5.HIV or X4.HIV for 2 d at different multiplicities of infection (MOIs) and then washed to remove input virus. Infection was quantified based on GFP expression at different time points after inoculation (Figure 5A), and viral replication was assessed by quantifying HIV p24 protein in culture supernatants. R5.HIV replicated efficiently in T_N_, T_CM_, and T_EMRO_ cells, but there was little or no replication in T_EMRA_ cells (Figure 5B). In contrast, X4.HIV infected and replicated efficiently in all four subsets and rapidly killed most of the T cells (Figure 5A and 5B, right panels; unpublished data). Similar results were observed when primary HIV-1 isolates, utilizing different R5-tropic, X4-tropic, and R5X4-dual tropic HIV-1 envelopes that also express *nef,* were used (Figure 5C). The infectivity of T_EMRA_ cells activated with SEB-pulsed DCs also remained identical (unpublished data).

**Figure 6:**
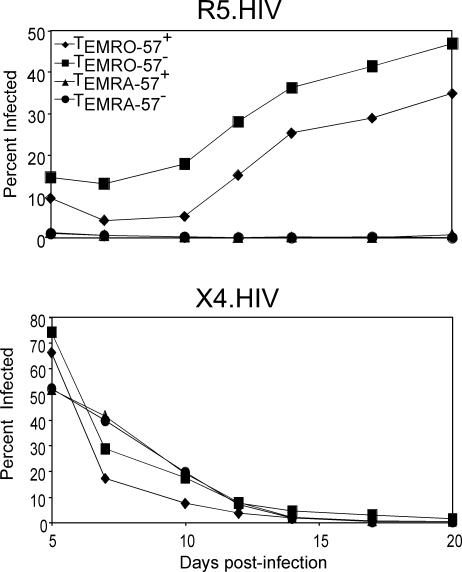
Replication of HIV-1 strains in CD57^+^ CD4^+^ T cell subsets Effector memory T cell subsets were subdivided into CD57^+^ and CD57^−^ portions by sorting, then activated and concurrently infected with R5.HIV or X4.HIV. The percentage of infected cells was determined by GFP expression at different time points post-infection by flow cytometry. The results are representative of one of five independent experiments from different individuals.

The surface marker CD57 identifies terminally differentiated cells [23], and expansion of CD57^+^ cells occurs in HIV-infected individuals [24]. Because T_EMRA_ cells were enriched in CD57^+^ cells (Figure 2), we asked whether CD57^+^ T cells were differentially susceptible to R5-tropic or X4-tropic viruses. For this experiment, T_EMRA_ and T_EMRO_ cells were further subdivided into CD57^+^ and CD57^−^ subsets by flow cytometry cell sorting. Both CD57^+^ and CD57^−^ subsets of T_EMRA_ cells were resistant to infection by R5-tropic virus, whereas both CD57^+^ and CD57^−^ subsets of T_EMRO_ cells remained susceptible to R5-tropic virus infection (Figure 6, top panel). However, the CD57^+^ and CD57^−^ subsets of both T_EMRO_ and T_EMRA_ cells were similarly susceptible to X4-tropic viruses (Figure 6, bottom panel). Thus, the relative resistance of T_EMRA_ cells to R5-tropic HIV was not attributable to enrichment with the CD57^+^ subset.

**Figure 7:**
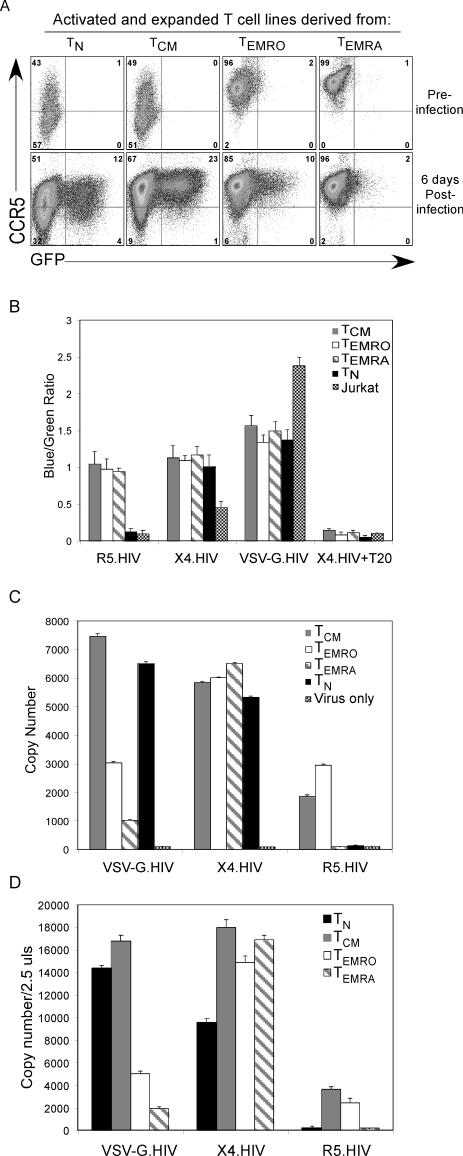
Identifying the Block in R5-Tropic and VSV-G Pseudotyped Infection of T_EMRA_ Cells Purified CD4^+^ T cell subsets were activated using DCs pulsed with SEB (10 ng/ml) and expanded in IL-2–containing medium for 10 d. (A) Day 12 expanded subsets were stained for CCR5 expression. Cells were then reactivated with SEB-pulsed DCs and subsequently infected with R5.HIV at an MOI of 5. At day 6 post-infection, cells were then stained for CCR5, and infection was analyzed as determined by GFP expression by flow cytometry. (B) R5.HIV, X4.HIV, and VSV-G.HIV carrying a β-lactamase reporter protein were incubated with expanded CD4^+^ T cell subsets, T_N_ cells, and Jurkat T cell line at 37 °C for 2 h to allow virus–cell fusion, CCF2/AM (20 μM) was added, and fluorescence was measured as described in Materials and Methods. Fluorescence ratios were calculated after subtraction of the average background fluorescence. (C) Expanded subsets were reactivated with SEB-pulsed DCs and subsequently infected with R5.HIV, X4.HIV, or VSV-G.HIV at an MOI of 5 for 12 h. Cells were then lysed and processed for real-time PCR analysis using late HIV transcript primers. (D) Reactivated expanded T cell subsets were lysed and processed for real-time PCR analysis using early HIV transcript primers. All infections were performed at an MOI of 5. The results are representative of one of five independent experiments using T cell subsets isolated from five different individuals.

We next investigated where in the HIV-1 life cycle R5-tropic infection of T_EMRA_ cells was blocked. Because large numbers of cells were required for these experiments, we expanded T_N_, T_CM_, T_EMRO_, and T_EMRA_ cells purified from PBMCs of HIV-uninfected individuals using SEB-pulsed DCs for 12 d in IL-2–containing medium. In order to verify that CCR5 expression levels were maintained on expanded T cell subsets and that T_EMRA_ cells remained resistant to R5-tropic infection, CCR5 expression was determined post-activation and expansion (Figure 7A, top panel). The expanded subsets were then reactivated with SEB-pulsed DCs and subsequently infected with R5.HIV. Although the T_EMRA_ cells maintained very high CCR5 expression, they remained resistant to R5-tropic infection (Figure 7A, bottom panel).

We first asked whether the block of R5-tropic infection was at the level of fusion. For this experiment, we employed a recently developed reporter assay to quantify HIV particle entry [25]. Expanded T_CM_, T_EMRO_, and T_EMRA_ cells were infected with either R5.HIV, X4.HIV, or VSV-G.HIV. Fusion of these three viruses with T_EMRA_ cells was similarly efficient, whereas fusion was inhibited in both T_N_ and Jurkat cells, which do not express CCR5, or when cells were pre-treated with T20, a fusion inhibitor (Figure 7B). Collectively, these data indicate that the R5.HIV infection block in T_EMRA_ cells is post-fusion.

We next conducted analysis of the stage in the HIV-1 life cycle at which R5-tropic and VSV-G pseudotyped virus replication was blocked in the T_EMRA_ subset. Late reverse transcripts in cells infected with VSV-G.HIV, R5.HIV, and X4.HIV were analyzed. Infection was blocked at the level of reverse transcription in T_EMRA_ cells infected with R5.HIV and VSV-G.HIV, suggesting an early block to infection in these cells that did not affect X4.HIV (Figure 7C).

Because we did not see the accumulation of late reverse transcription products, we wanted to understand whether earlier steps in reverse transcription were impaired. Therefore, we investigated the initiation of reverse transcription of R5-tropic and VSV-G pseudotyped virus in T_EMRA_ cells. Early reverse transcription was assessed by the presence of strong-stop, minus-strand viral DNA (R/U5 DNA) by quantitative real-time PCR. Early transcripts were not formed in T_EMRA_ cells infected with R5.HIV or VSV-G.HIV (Figure 7D). These data suggest that the block in the HIV-1 life cycle occurs at or prior to the initiation of reverse transcription.

### Correlation of T_EMRA_ Cell Levels with CD4^+^ Cell Numbers in HIV-Infected Individuals

**Figure 8:**
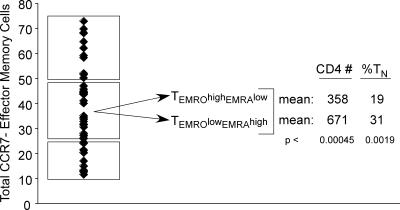
Association between T_EM_ Subsets and CD4 Numbers in HIV-Infected Individuals HIV-infected individuals were separated into three groups based on percent of their CD4^+^ T_EM_ cells. The medium T_EM_ population was subdivided into T_EMRO_^high^T_EMRA_^low^ and T_EMRO_^low^T_EMRA_^high^, and the number of total CD4 cells and T_N_ cells in each group was calculated. We set the cut-off percentage for defining T_EMRO_ high and low proportions as 28% of CD4^+^ T cells and for T_EMRA_ cells, 8% of CD4^+^ T cells. Statistical analysis was performed with only the medium group since the bottom group did not contain any T_EMRA_^high^ cells (range: 1.6%–5.5%) and within the top group all individuals contained high T_EMRO_ cells (range: 31%–59%). Statistical significance between groups was determined by Wilcoxon rank sum test.

Our findings that T_EMRA_ cells are expanded in a portion of HIV-infected individuals and are highly resistant to R5-tropic infection prompted us to examine relationships between high T_EMRA_ cells and CD4 numbers. Among HIV-infected individuals, the T_N_ cell percentage correlated positively with absolute CD4^+^ T cell numbers (Figure 1B). Conversely, the total T_EM_ cell (T_EMRO_ + T_EMRA_) percentage correlated negatively with CD4^+^ T cell numbers (Figure 1B; unpublished data). To further delineate the association between T_EMRO_ and T_EMRA_ cell proportions and CD4 numbers, we subdivided infected individuals into three groups based on their total T_EM_ cells (Figure 8). Infected individuals in whom the T_EM_ percentage of their CD4^+^ T cells was similar to healthy individuals (bottom group) had high CD4^+^ cell numbers (Figure 8; unpublished data). In contrast, the group with a very high T_EM_ cell percentage had low CD4^+^ T cell numbers, and all of these individuals had high levels of T_EMRO_ cells (Figure 8, top group). Importantly, however, when we subdivided the infected individuals with median levels of T_EM_ cells (Figure 8, middle group), a highly significant association between higher T_EMRA_ cell percentage and higher CD4^+^ T cell numbers and higher T_N_ cells was established (Figure 8). These results imply that a greater proportion of T_EMRA_ cells within the effector T cell subset may identify individuals with better preservation of CD4^+^ cell numbers, and possibly slow HIV-1 disease progression.

## Discussion

Our investigation of memory T cell subsets during HIV-1 infection led to the discovery of a unique subset of CD4^+^ T cells called CD4^+^ T_EMRA_ cells. We found that these cells are highly susceptible to infection by X4-tropic HIV-1 but are almost completely resistant to R5-tropic HIV-1 despite high levels of cell surface CCR5 expression. These cells are also relatively resistant to infection by VSV-G pseudotyped HIV-1. Our findings are consistent with a recent ex vivo analysis of T cell subsets from HIV-infected individuals, which demonstrated that CD4^+^CD57^+^ effector memory T cells were associated with approximately ten times fewer copies of viral DNA than T_CM_ cells [23]. Although both CD57^+^ and CD57^−^ subsets of T_EMRA_ cells displayed the same R5-tropic HIV-1 infection (Figure 5C), overall, CD57^+^ cells are more enriched within T_EMRA_ cells (Figure 2). Thus, T_EMRA_ cells represent the first unique subpopulation of CD4+ T cells that are uniquely resistant to HIV-1 infection and may emerge as a consequence selection during infection.

Further studies are required to elucidate how T_EMRA_ cells can be resistant to R5-tropic infection despite high levels of CCR5 expression, yet remain susceptible to X4-tropic viruses. In order to exclude that this restriction was at the level of post-entry and not because of downregulation or block of CCR5 by beta-chemokines, we showed that 1) T_EMRA_ cells permitted entry of R5-tropic HIV-1 as measured by the BlaM-Vpr virion fusion assay, 2) T_EMRA_ cells continued to express high levels of CCR5 at the time of infection, 3) and T_EMRA_ cells were partly less susceptible to VSV-G pseudotyped viruses that bypass the coreceptor requirement. Taken together, these results indicate that the post-entry pathway followed by R5-tropic HIV-1 may differ in T_EMRA_ cells compared to other CD4^+^CCR5^+^ T cell subsets and to X4-tropic HIV-1–infecting T_EMRA_ cells. It is conceivable that either signaling or the entry pathway through the CXCR4 receptor elicits intracellular events needed for HIV replication or bypasses mechanisms that otherwise restrict HIV-1 in T_EMRA_ cells.

Elucidating cellular mechanisms that determine why some, but not all, CCR5-expressing CD4^+^ T cells are permissive to R5-tropic HIV-1 infection could provide clues to identify natural cellular HIV-1 barriers. Our findings suggest that at least one subset of primary human T cells display intrinsic restriction that limits HIV-1 infection. The presence of differentiated T_EMRA_ cells in HIV-1 infected individuals and in uninfected individuals, albeit at lower frequency, suggests that these cells expand and survive during the course of the normal immune response. These findings also pose several important questions: How do T_EMRA_ cells arise? Are they repeatedly stimulated memory T cells? What aspect of the T_EMRA_ cell differentiation program renders them resistant to HIV-1 infection? For example, T_EMRA_ cells displayed a preferential Th1 phenotype and exhibited a reduced proliferative capacity as well as a cell surface marker and cytokine profile characteristic of highly differentiated T cells. A subset of CD8^+^ T cells that are CD45RA^+^CD27^−^ (CD8^+^ T_EMRA_ cells) has been shown to display similar phenotypic features to CD4^+^ T_EMRA_ cells characterized here [8,20,26]. It is not yet clear whether CD4^+^ T_EMRA_ cells are functionally similar to CD8^+^ T_EMRA_ cells or what role these subsets play during chronic viral infections. The homeostatic mechanisms that induce and maintain CD4^+^ T_EMRA_ cells also remains to be determined.

Our finding that T_EMRA_ cells correlate with higher CD4^+^ T cell numbers in a portion of HIV-infected individuals suggests that virus infection may positively drive selection for HIV-resistant cells in vivo, a phenomenon previously observed only in cell culture but usually involving loss of CD4 expression. Studies using animal models for HIV-1 infection may aid in determining whether there is a causal relationship between virus infection and selective enrichment of the T_EMRA_ subset. Remarkably, HIV-infected individuals whose T_EM_ cells were composed mostly of T_EMRA_ cells were significantly associated with higher CD4^+^ T cell and T_N_ cell levels. How T_EMRA_ cells accumulate or expand in HIV patients, and whether they have a protective role against progression of disease, remains to be determined. Memory and effector T cells are enriched for CCR5 expression [17,18], suggesting that they are targets for HIV-1, especially T cells resident in the gut tissue [27–30]. It is conceivable that after continuous destruction of susceptible T_EMRO_ cells, an HIV-resistant subset of T_EMRA_ cells is selected. Alternatively, T_EMRA_ cells may have a protective role against HIV-1 infection, perhaps because HIV-specific T cells are enriched in this subset. If T_EMRA_ cells contain a high proportion of HIV-specific effector T cells, this would overcome a potential Achilles' heel of the immune response during HIV-1 infection; that is, CD4^+^ T cells that are activated by HIV-1 antigens themselves become highly susceptible targets for the virus [31]. Conferring an HIV-resistant ability to HIV-1–specific CD4^+^ T cells could lead to novel strategies aimed at potentiating a protective immune response against HIV-1 infection.

During the primary and asymptomatic phases of HIV-1 infection, R5-tropic viruses predominate, whereas X4-tropic viruses are found in about 50% of infected individuals at late stages of HIV disease [32–34]. A more rapid decline in total CD4^+^ T cell counts is often associated with a switch from R5-tropic to X4-tropic HIV or R5/X4 HIV variants [35]. At present, it is unclear whether the switch to X4-tropic viruses is a cause or a consequence of the collapse of the immune system. Because T_EMRA_ cells remain highly susceptible to X4-tropic viruses, it would be expected that these cells would also be rapidly depleted when an X4-tropic switch occurs. If T_EMRA_ cells contain HIV-specific T cells or play some other protective role against infection, then elimination of these cells by X4-tropic viruses would further weaken the immune response against HIV-1 and facilitate immunological deterioration.

In summary, our results demonstrate that CD4^+^ T_EMRA_ cells are present at a higher frequency in HIV-infected than uninfected individuals and are resistant to R5-tropic HIV infection, but not to X4-tropic HIV-1 infection. Studies focused on emergence of these effector memory T cell subsets will contribute to a better understanding of HIV-1 pathogenesis and the role of these cells during normal immune responses. Decoding the precise molecular mechanism of the intrinsic resistance of T_EMRA_ cells to R5-tropic infection may have significant implications for developing novel approaches to endow this unique phenotype on HIV-1–susceptible T cells.

## Materials and Methods

### Cell isolation and culture.

PBMCs were separated from blood of HIV-uninfected and HIV-infected individuals through Ficoll-Hypaque (Pharmacia, http://www.pfizer.com). Resting CD4^+^ T cells were purified as previously described [22] and were at least 99.5% pure as determined by post-purification FACS analysis. To purify naïve, central, and effector memory subsets, purified CD4^+^ cells were stained with CCR7 and CD45RO antibodies, and CD45RO^−^CCR7^+^ (T_N_), CD45RO^+^CCR7^+^ (T_CM_), CD45RO^+^CCR7^−^ (T_EMRO_), and CD45RO^−^CCR7^−^ (T_EMRA_) subsets were sorted using the flow cytometer (FACSAria; BD Biosciences, http://www.bdbiosciences.com). The culture medium used in all experiments was RPMI (Cellgro, http://www.cellgro.com) and prepared as described before [22]. All cytokines were purchased from R&D Systems (http://www.rndsystems.com). In some experiments, T_EMRO_ and T_EMRA_ subsets were further subdivided into CD57^+^ and CD57^−^ subsets by flow sorting by staining purified CD4^+^ T cells with CCR7, CD45RO, and CD57 antibodies. Monocyte-derived DCs were generated as previously described [22]. The superantigen SEB (Sigma, http://www.sigmaaldrich.com) was used to stimulate resting T cells in the presence of DCs [36].

### Study participants.

Uninfected individuals were adults (ages 21–64, mean age was 32) with no history of HIV infection. Whole blood samples from adult participants with HIV infection were obtained during routine primary care visits. Among the HIV-infected individuals, 76% were Caucasian, 82% were male, the median (range) age was 41 (28–59) years, and 79% were receiving potent antiretroviral therapy. Median (IQR) CD4^+^ T cell and log_10_ plasma HIV-1 RNA values were 380 (270–592) cells/mm^3^ and 2.7 (2.6–3.8) copies/ml plasma, respectively, and 50% had fewer than 400 HIV-1 RNA copies/ml in plasma. There were no selection criteria based on race or sex. All participants provided written informed consent that was approved by the Vanderbilt Institutional Review Board.

### Virus production and infections.

VSV-G pseudotyped replication-incompetent HIV were generated as previously described [36]. R5-tropic and X4-tropic replication-competent viruses were prepared similarly by transfecting 293T cells with HIV that encodes either R5-tropic (BaL) or X4-tropic (NL4–3) envelope and EGFP (Clontech, http://www.clontech.com) in place of the *nef* gene as previously described [37]. Wild-type virus (NL4–3) with X4-tropic or with R5-tropic envelope (BaL) and virus (R8) encoding heat stable antigen (HSA) in place of *vpr* [38] with intact *nef* gene were obtained from Chris Aiken (Vanderbilt University). Additional viruses used in this study were as follows. NL4–3–based proviral constructs encoding Env genes from R5-tropic proviral 92MW965.26, NL JRFL, NL YU2, and dual-tropic NL89.6 were obtained from Paul Bieniasz (Aaron Diamond AIDS Research Center) and have been previously described [39]. R5-tropic virus JRCSF and X4-tropic virus R9 were obtained from Vineet KewalRamani (National Cancer Institute [NCI]). Typically, viral titers ranged from 1 × 10^6^ to 5 × 10^6^ IFU/ml for replication-competent viruses and 10 × 10^6^ to 30 × 10^6^ IFU/ml for VSV-G pseudotyped HIV, as titered on CCR5-expressing Hut78 T cell lines (gift of Vineet KewalRamani, NCI). Viral replication in T cell cultures was determined by measuring p24 levels within supernatants by an ELISA.

### T cell apoptosis and cytokine assays.

To determine apoptosis, T cells were stimulated with α-CD3 (OKT-3, ATCC)–coated plates in the presence of soluble α-CD28 (1 μg/ml; Pharmingen, http://www.bdbiosciences.com) for 18 h. T cell apoptosis was measured by PE-conjugated Annexin V according to manufacturer's instructions (BD Biosciences). Cytokines (IL-2, IL-4, IL-5, IL-10, TNF-α, IFN- γ) in the supernatants were assayed using a commercially available cytometric bead assay (CBA) (BD Biosciences) [40], and analyzed using CBA 6-bead analysis software (BD Biosciences).

### FACS analysis.

T cells were stained with the relevant antibody on ice for 30 min (chemokine receptor staining performed at room temperature for 20 min) in PBS buffer containing 2% FCS and 0.1% sodium azide. Cells were then washed twice, fixed with 1% paraformaldehyde, and analyzed with a FACSCalibur or FACSAria flow cytometer. Live cells were gated based on forward and side scatter properties, and analysis was performed using FlowJo software (Tree Star, http://www.treestar.com). The following anti-human antibodies were used for staining: CD3, CD4, CD8, CD45RO, CD45RA, CD28, CD27, CD11b, CD57, CD7, CD62L, HLA-DR, CCR5 (all from BD Biosciences), CCR7, and CCR4 (R&D Systems). The CRTH2 antibody used for these experiments has been previously described [41]. Secondary goat-anti-mouse antibodies were conjugated with allophycocyanin or PE (BD Biosciences). For the intracellular p24 stain, fixation and permeabilization was performed using a commercial kit (BD Biosciences) according to the manufacturer's instructions. Subsequently, cells were stained with anti-p24 for 1 h, followed by goat-anti-mouse conjugated to allophycocyanin for 30 min.

### Virus–cell fusion assay.

HIV fusion assays were performed essentially as previously described [25]. Briefly, viruses carrying a β-lactamase reporter protein fused to the amino terminus of the virion protein Vpr (BlaM-Vpr) were added to expanded T cell subsets at 37 °C for 2 h to allow virus–cell fusion. CCF2/AM (20 μM; Aurora Biosciences Corporation, http://www.vrtx.com) was added, and the cultures were incubated for 14 h at room temperature. Cells were pelleted and resuspended in PBS, and the fluorescence was measured at 447 and 520 nm with a microplate fluorometer after excitation at 409 nm. Uncleaved CCF2 fluoresces green, due to fluorescence resonance energy transfer between the coumarin and fluorescein groups; however, cleavage by BlaM results in the dissociation of these fluorophores, and the emission spectrum shifts to blue. Thus, the ratio of blue to green cellular fluorescence is proportional to the overall extent of virus–cell fusion. Fluorescence ratios were calculated after subtraction of the average background fluorescence of control cultures containing no virus (blue values) and wells containing PBS (green values).

### Quantitative analysis of HIV reverse transcription in target cells.

Viral DNA was quantified by real-time PCR using an ABI 7700 instrument (PE Biosystems, http://www.appliedbiosystems.com) with SYBR Green chemistry. The reaction mixtures (25 μl total volume) contained 2.5 μl of infected lysate, 12.5 μl of 2x SYBR Green PCR Master Mix (PE Biosystems), and 50 nM of each primer. A standard curve was prepared from serial dilutions of HIV plasmid DNA. The reactions were amplified and analyzed as previously described [42]. The sequences of primers (R and U5) specific for early products were 5′-GGCTAACTAGGGAACCCACTGCTT (forward) and 5′-CTGCTAGAGATTTTCCACACTGAC (reverse). The late-product primer sequences (R and 5NC) were 5′-TGTGTGCCCGTCTGTTGTGT (forward) and 5′-GAGTCCTGCGTCGAGAGAGC (reverse), as previously described.

### Statistical analysis.

Statistical analyses were performed using Stata version 9.0 (http://www.stata.com). T cell subset and clinical data are presented as means (standard deviation). Statistical significance between groups was determined by Wilcoxon rank sum test. Differences were considered significant at *p* < 0.05.

## Supporting Information

Figure S1 CRTH2 Expression on CD4^+^ T Cell Subsets Purified CD4^+^ T cells were stained with CD45RO and CCR7 in conjunction with the CRTH2 antibody. Electronic gates were set on T_N_, T_CM_, T_EMRO_, and T_EMRA_ cells as described in Figure 1, and expression of CRTH2 was analyzed by flow cytometry. The results show mean expression from ten different individuals.
(146 KB TIF)

Figure S2 CCR5 Expression on CD4^+^ T Cell Subsets PBMCs from HIV-positive individuals were stained with CD4, CD45RO, CCR7, and CCR5 antibodies. Electronic gates were set on CD4^+^ T_N_, T_CM_, T_EMRO_, and T_EMRA_ cells as described in Figure 1, and expression of CCR5 was analyzed by flow cytometry. The results show CCR5 expression from 20 different individuals. The median CCR5 expression levels were: T_N_ = 0.5, T_CM_ = 11.96, T_EMRO_ = 46, and T_EMRA_ = 25.1. Statistical significance was determined for T_EMRO_ and T_EMRA_ versus T_CM_ using the Student's two-tailed *t* test. * *p* < 0.05.
(174 KB TIF)

## Abbreviations

DC: dendritic cell
GFP: green fluorescent protein
PBMC: peripheral blood mononuclear cell
R5.HIV: CCR5-tropic replication-competent HIV
SEB: staphylococcal enterotoxin B
T_CM_: central memory T cell
TCR: T cell receptor
T_EM_: effector memory T cell
T_EMRA_: effector memory CD45RA^+^ T cell
T_EMRO_: effector memory CD45RA^−^ T cell
T_N_: naïve T cell
VSV-G.HIV: vesicular stomatitis virus glycoprotein G pseudotyped HIV
X4.HIV: CXCR4-tropic replication-competent HIV
