# Supplementary figures and images for: Identification of a CCR5-Expressing T Cell Subset That Is Resistant to R5-Tropic HIV Infection
Source: PLoS Pathog. 2007 Apr 27;3(4):e58. doi: 10.1371/journal.ppat.0030058 (PMC1857714; doi:10.1371/journal.ppat.0030058)

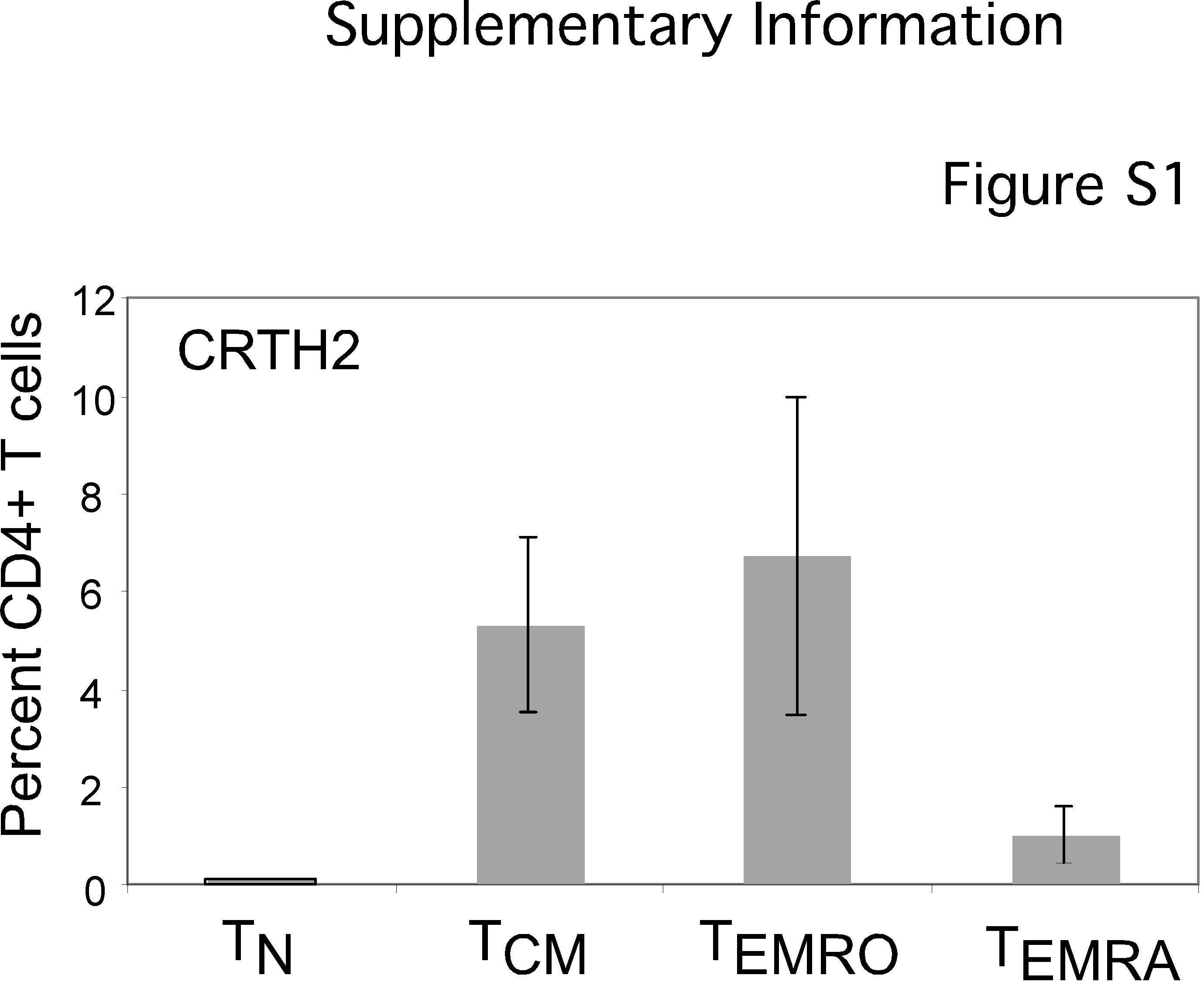

Supplement: Figure S1 — Purified CD4+ T cells were stained with CD45RO and CCR7 in conjunction with the CRTH2 antibody. Electronic gates were set on TN, TCM, TEMRO, and TEMRA cells as described in Figure 1, and expression of CRTH2 was analyzed by flow cytometry. The results show mean expression from ten different individuals. (146 KB TIF) [file ppat.0030058.sg001.tif]

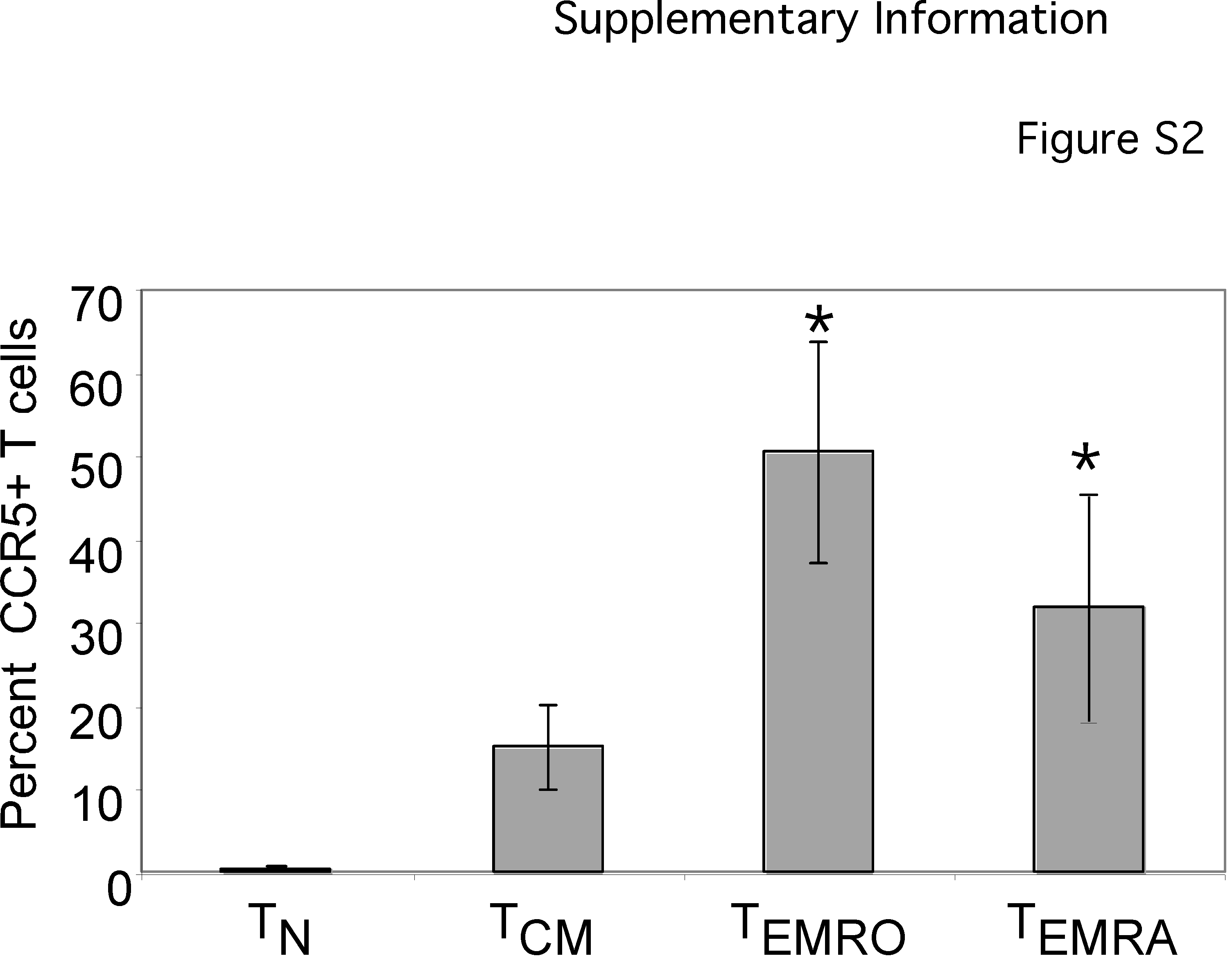

Supplement: Figure S2 — PBMCs from HIV-positive individuals were stained with CD4, CD45RO, CCR7, and CCR5 antibodies. Electronic gates were set on CD4+ TN, TCM, TEMRO, and TEMRA cells as described in Figure 1, and expression of CCR5 was analyzed by flow cytometry. The results show CCR5 expression from 20 different individuals. The median CCR5 expression levels were: TN = 0.5, TCM = 11.96, TEMRO = 46, and TEMRA = 25.1. Statistical significance was determined for TEMRO and TEMRA versus TCM using the Student's two-tailed t test. * p < 0.05. (174 KB TIF) [file ppat.0030058.sg002.tif]
